# Microbial Ecology Pushes Frontiers in Biotechnology

**DOI:** 10.1264/jsme2.ME2901rh

**Published:** 2014-03

**Authors:** Atsushi Kouzuma, Kazuya Watanabe

**Affiliations:** 1 School of Life Sciences, Tokyo University of Pharmacy and Life Sciences, 1432–1 Horinouchi, Hachioji, Tokyo 192–0392, Japan

Due to their diverse metabolic potentials and established manipulation techniques, microbes are considered to be excellent materials for biotechnology. Pure cultures of microbes are exploited in industrial processes to produce alcohols, organic acids, and polymeric materials (2). Furthermore, human society has a long history of using mixed microbial cultures to produce fermented foods (8). Naturally occurring microbial communities are also used in various environmental biotechnologies (25), such as wastewater treatment, methanogenic digestion, bioremediation, and microbial fuel cells. The microbial communities involved in these processes have been the targets of intensive investigations because not only scientists, but also engineers are interested in how microbes work in such communities and/or how the performances of ecosystems are supported and influenced by individual microbes. Recent advances in microbial ecology have contributed to a better understanding of microbes and their activities in these processes, and provided the fundamentals for their future development. We herein focused on the latest research highlights in microbial ecology that are relevant to processes in environmental biotechnology.

Large volumes of wastewater are discharged due to the activities of human society, and most are treated by biological processes, such as activated-sludge processes. These processes exploit naturally occurring microbial communities, in which community structures are modified according to changes in the chemical compositions of wastewater and environmental conditions. Therefore, understanding microbes and their activities is considered important for the successful operation of these processes. Marked advances have recently been made in nucleotide-sequencing technologies, resulting in a new era of molecular and genomic approaches in microbial ecology (18, 28), including the pyrosequencing technique used to determine the nucleotide sequences of taxonomic marker-gene (*e.g.*, 16S rRNA genes) fragments PCR-amplified from community metagenomes (5). Sato *et al.* used pyrosequencing to monitor bacterial population dynamics in laboratory activated-sludge reactors (26), and acquired more than 80,000 sequences for analyzing bacterial populations. Sufficient phylogenetic knowledge could even be obtained for specific genus-level lineages, such as *Accumulibacter phosphatis*. This genus is known to include phosphate-accumulating organisms that play important roles in the removal of phosphorus in wastewater-treatment plants (7). These organisms also have the ability to accumulate biopolymers, such as polyhydroxyalkanoate (24).

Nitrogen removal is another important task in the process of wastewater treatment, and the microbes responsible have been the focus of recent studies. Aerobic tanks are used for nitrification, in which ammonia is oxidized to nitrite by ammonia-oxidizing microbes and is further oxidized to nitrate by nitrite-oxidizing microbes (1). Although molecular analyses have identified a large number of microbes that may be involved in this conversion (3), most of these microbes remain uncultured, and their activities and physiologies have not yet been sufficiently elucidated. Recent studies isolated new nitrifiers, which has expanded our knowledge on these biotechnologically important activities. For example, a thermotolerant ammonia-oxidizing bacterium was isolated from activated sludge in a thermal power station (13). Furthermore, nitrite-oxidizing *Nitrospira*, which is known to be abundant in activated sludge, but resistant to cultivation, have successfully been enriched (6) and isolated (29) from municipal wastewater-treatment plants. Another recent study documented the successful cultivation of planktonic anaerobic ammonia-oxidizing bacteria using membrane bioreactors (23). Cultivation is essential for analyzing the physiology of microbes, and these studies have also provided novel insights into the strategies used to cultivate and isolate various cultivation-resistant microbes in the environment.

Recent studies have led to important advances in our understanding of the microbes involved in the anaerobic methanogenic digestion of organic wastes. A previous study found novel lineages of methanogens from a thermophilic digester, for which the order *Methanomassiliicoccales* has been proposed (12). Another study identified an as-yet-unknown interspecies interaction, called “electric syntrophy”, which has been shown to accelerate methanogenesis by rapid interspecies electron transfer via electric currents through (semi)conductive iron-oxide minerals (15). Since interspecies electron transfer between fermentative bacteria and methanogenic archaea is considered to be the bottleneck step in methanogenesis, these findings are appreciated by engineers who have attempted to develop stable and efficient anaerobic digesters.

Microbial fuel cells (MFCs) are devices that use living microbes as catalysts for the conversion of fuels (*e.g.*, organic compounds) into electricity, and have recently attracted attention as sustainable bioenergy systems (31). The diverse catabolic activities of bacteria provide MFCs that have advantages over chemical fuel cells, which can only utilize purified reactive fuels (*e.g.*, hydrogen). In addition, MFCs employing naturally occurring microbial communities were shown to be capable of generating electricity from waste biomass and wastewater (21, 27). In MFCs, electrochemically active bacteria (EAB), which are able to transfer electrons to extracellular solid acceptors, play key roles in electricity generation. Many EAB have been isolated and characterized from MFCs and natural environments (32, 33). Previous studies also demonstrated that EAB played critical roles in the geochemical cycling of metals in the environment. Recent studies have found that soil bacteria transfer electrons via electric currents through conductive iron-oxide minerals (16). The importance of conductive mineral particles for the physiology of EAB has also been suggested (17). A better understanding of the physiology and ecology of EAB will contribute to the future development of efficient MFCs, thereby facilitating their practical application.

Bioremediation is a process that utilizes microbes to clean polluted environments. Various microorganisms that exhibit the potential to degrade and detoxify various types of pollutants, including xenobiotic compounds and toxic metals, have been isolated (20, 30). In addition, recent advances in molecular and genomic techniques, including metagenomics and metatranscriptomics, have accelerated the understanding of metabolic pathways and genetic functions underlying bioremediation processes (4). The importance of interspecies interactions has been demonstrated among biodegradative microbes. For example, the findings of recent studies have indicated that the reductive dechlorination of polychlorinated dipenzo-p-dioxins and oxidative degradation of dechlorinated products occur simultaneously in semi-anaerobic microbial microcosms, which facilitates their complete dechlorination (11, 14).

Microorganisms also have the potential to remove various toxic metals from contaminated environments by altering their chemical speciation and oxidation states. For example, some dissimilatory metal-reducing bacteria are known to possess the ability to reduce the soluble oxidized form of uranium, U(VI), to insoluble U(IV), thereby preventing from their further spread to uncontaminated sites (10). Recent studies also characterized U-tolerant bacterial communities in subsurface U ore deposits (19). On the other hand, microorganisms that can reduce arsenate [As(V)] to arsenite [As(III)], which has greater toxicity and hydrological mobility than arsenate, have also been discovered, and the molecular mechanisms responsible for arsenate reduction have been identified(22). In addition, studies reported the isolation and characterization of As(III)-and antimonite [Sb(III)]-oxidizing bacteria that potentially contribute to the speciation and mobility of Sb and As *in situ* (9). Although these processes have yet to be practically applied to large-scale environmental restoration, a better understanding of microbe-metal interactions will contribute to the search for new remediation strategies.

We herein introduced recent highlights in microbial ecology that are relevant to wastewater treatment, anaerobic digestion, microbial fuel cells, and bioremediation. Although the engineering techniques used and microbes involved in these processes are different, microbial ecology is the common science that provides an interdisciplinary understanding of the microbes involved. On the other hand, we suggest that these processes serve as model ecosystems that can be used to assess novel theories in microbial ecology. Therefore, environmental biotechnology and microbial ecology are expected to co-evolve based on technical advancements and social needs.
